# Canine Evolution in Sabretoothed Carnivores: Natural Selection or Sexual Selection?

**DOI:** 10.1371/journal.pone.0072868

**Published:** 2013-08-08

**Authors:** Marcela Randau, Chris Carbone, Samuel T. Turvey

**Affiliations:** Institute of Zoology, Zoological Society of London, Regent’s Park, London, United Kingdom; Monash University, Australia

## Abstract

The remarkable elongated upper canines of extinct sabretoothed carnivorous mammals have been the subject of considerable speculation on their adaptive function, but the absence of living analogues prevents any direct inference about their evolution. We analysed scaling relationships of the upper canines of 20 sabretoothed feliform carnivores (Nimravidae, Barbourofelidae, Machairodontinae), representing both dirk-toothed and scimitar-toothed sabretooth ecomorphs, and 33 non-sabretoothed felids in relation to body size in order to characterize and identify the evolutionary processes driving their development, using the scaling relationships of carnassial teeth in both groups as a control. Carnassials display isometric allometry in both sabretooths and non-sabretooths, supporting their close relationship with meat-slicing, whereas the upper canines of both groups display positive allometry with body size. Whereas there is no statistical difference in allometry of upper canine height between dirk-toothed and scimitar-toothed sabretooth ecomorphs, the significantly stronger positive allometry of upper canine height shown by sabretooths as a whole compared to non-sabretooths reveals that different processes drove canine evolution in these groups. Although sabretoothed canines must still have been effective for prey capture and processing by hypercarnivorous predators, canine morphology in these extinct carnivores was likely to have been driven to a greater extent by sexual selection than in non-sabretooths. Scaling relationships therefore indicate the probable importance of sexual selection in the evolution of the hypertrophied sabretooth anterior dentition.

## Introduction

The elongated upper canines of sabretoothed carnivores represent a classic example of a highly specialized morphological trait that has evolved repeatedly in the past, but the function of which is still not fully understood due to a lack of close analogues in living species. This morphotype has appeared in nimravids, barbourofelids and machairodonts (Carnivora: Feliformia), as well as some other carnivorous placentals (e.g. *Machaeroides*: Creodonta), marsupials (*Thylacosmilus*: Borhyaenidae) and non-mammalian therapsids (e.g. gorgonopsids) [1,2]. However, the hypertrophied sabretooth condition is only approached today in mammalian carnivores by clouded leopards ( *Neofelis* ), with other living felids having shorter, conical-shaped upper canines [3].

There has been considerable scientific interest and debate about the function and evolution of sabretooth canines [4–7], but establishing the relationship between form and function when interpreting morphological traits in extinct species is a perennial challenge in palaeobiology [8,9]. Among extant mammalian carnivores, canines play an important role in prey-killing behaviour, and show considerable variability associated with variation in prey resource base and presence/absence of ecological competitors [10]. Sabretooth skeletal and dental morphology suggests a hypercarnivorous habit (i.e. >70% vertebrate prey) consistent with the need for well-developed anterior dentition [2,4–7,11], but the extremely exaggerated morphology of sabretoothed canines may have served an additional or alternative function. The evolution of ‘bizarre’ morphological structures is often associated with sexual selection, where they can represent ornaments to attract mates (intersexual selection) or weapons to combat rivals (intrasexual selection) [12,13]. Elongated canines associated with sexual selection rather than food acquisition/processing are found in males of several deer and primate genera (e.g. *Hydropotes* , *Moschus* , *Muntiacus* , *Papio*) [14,15], as well as other extinct mammals (e.g. dinoceratans) and non-mammalian therapsids [15]. However, because there are no true sabretoothed mammalian carnivores today, we cannot determine their predatory or social behaviour [16] or, importantly, whether their unusual canine morphology evolved for hunting or other functions. Furthermore, sabretoothed feliforms were not a morphologically homogenous group but instead consisted of three different recognised ecomorphs: (1) dirk-tooths (e.g. *Barbourofelis*, *Eusmilus*, *Hoplophoneus*, *Megantereon*, *Paramachairodus*, *Smilodon*), characterized by very elongated and laterally compressed canines, relatively shorter incisors, a long sagittal crest, and a robust, almost bear-like body shape; (2) scimitar-tooths (e.g. *Dinictis*, *Homotherium*, *Ischyrosmilus*, *Machairodus*, *Nimravides*, *Nimravus*), characterized by shorter, less compressed and often coarsely serrated canines, longer incisors, a shorter sagittal crest/temporalis musculature associated with relatively weaker bite force, and a more felid-like body shape; and (3) *Xenosmilushodsonae* , which had a robust body and increased sagittal crest but relatively short canines [17,18]. These ecomorphs are thought to be associated with different hunting strategies (ambush predation in dirk-tooths versus cursorial predation in scimitar-tooths [17]), and it is therefore possible that different evolutionary pressures may have acted specifically on the upper canines of each ecomorph, further complicating interpretation of the function of the ‘generalized’ sabretoothed morphotype.

The function of specific traits has often been inferred from the magnitude of the scaling exponent of a power function, b [12]. In particular, sexually selected ornaments and weapons frequently show positive allometry with body size dimensions (*b*>1 for comparisons between measures with the same dimensions, e.g. length–length), due to differential resource allocation to structures that enhance mating success. Slopes as high as 3.4, with a mode between 1.5–2.0, occur in traits associated with sexual display (e.g. antler size in the giant deer *Megaloceros*), possibly for reasons such as the “handicap principle” [12,13,19,20] whereby traits that are supposedly disadvantageous, and therefore costly to maintain, advertise for ‘better’ quality of individuals that can maintain them. On the other hand, traits that have evolved under natural rather than sexual selection often scale isometrically against body size (i.e. geometric similarity, slope=1), but in some cases can also show positive or negative allometries [12,13,20,21].

Although sexual selection has previously been suggested as a possible explanation for the hypertrophied sabretooth anterior dentition [22], this hypothesis has not previously been investigated through comparative analysis. Here we investigate the evolutionary drivers of the sabretooth morphotype in mammalian carnivores through comparison of allometric relationships between tooth size and body size in extinct sabretoothed feliforms and non-sabretoothed felids. The conical upper canines of living felids are thought to have evolved in association with prey-killing behaviour, and have therefore been subjected to strong natural selection [2,10]. If the sabretooth morphotype also evolved primarily for hunting, we would predict a similar scaling relationship between canine height and body size in both sabretooths and non-sabretooths, although with a different intercept. A steeper allometric slope in sabretooths would instead be consistent with the hypothesis that the sabretooth morphotype was sexually selected and associated with some form of display function, rather than being only or primarily associated with hunting. We also further investigate scaling relationships between dirk-toothed and scimitar-toothed sabretooth ecomorphs, to assess whether patterns of natural selection versus sexual selection were consistent across the evolutionary history of all sabretoothed feliforms; it is not possible to investigate scaling relationships for the third sabretooth ecomorph, as this only comprises a single taxon, *Xenosmilus* [17], making a multi-species regression impossible. Finally, we compare measurements of the carnassials (P4 and m1), shearing cheek teeth that are not externally visible and therefore unlikely to function in display behaviour. Although sabretooths had larger carnassials than non-sabretoothed felids [3], this would affect the intercept but not the slope of the scaling relationship for carnassials between these two carnivore groups. However, if carnassials and canines show different scaling patterns within specific groups, then this would also represent evidence for a display function for the anterior dentition in these groups.

## Materials and Methods

We compared morphometric data on 20 species representing all three taxonomic groups of extinct sabretoothed feliforms (Nimravidae, Barbourofelidae, Machairodontinae) and both of the main sabretooth ecomorphs (dirk-toothed and scimitar-toothed) with 33 species of non-sabretoothed extant and extinct felids, which represent the morphologically and phylogenetically closest available taxonomic control group [1]. For all species, we obtained measurements of tooth morphology (upper canine height, CH, from tooth tip to dentin-enamel junction; upper canine mediolateral width, CW; upper canine anteroposterior length, CL, following gum line; upper carnassial anteroposterior length, P4L; lower carnassial anteroposterior length, m1L; all measurements following [23]), and condylobasal skull length, SL, a measure of total body size; all measurements in mm (Electronic Supplementary Material Table S1). Direct body mass measurements were not used, because estimates calculated from skeletal measurements using predictive regression equations are unavailable for most extinct species. Most measurement data were collected from the published literature, with further measurements obtained from undamaged craniodental specimens in the Natural History Museum, London, using digital calipers (measured to nearest 0.01 mm), or from unpublished data (Graham S. Slater and Julie Meachen-Samuels, personal communication 2010). Measurement means were taken when multiple measurements were available per species. All data were log-transformed for analysis.

Dental measurements were regressed against SL in both sabretooths and non-sabretoothed felids, with regression slopes compared between the two groups. Analyses were performed using the Standardised Major Axis (SMA) calculation from the ‘smatr’ package in R [24]; SMA analyses are able to summarize the relationship between two variables without necessarily predicting one from the other, and are the most recommended approach when testing for isometry [25]. A normal distribution was tested and confirmed for all data.

## Results

**Figure 1:**
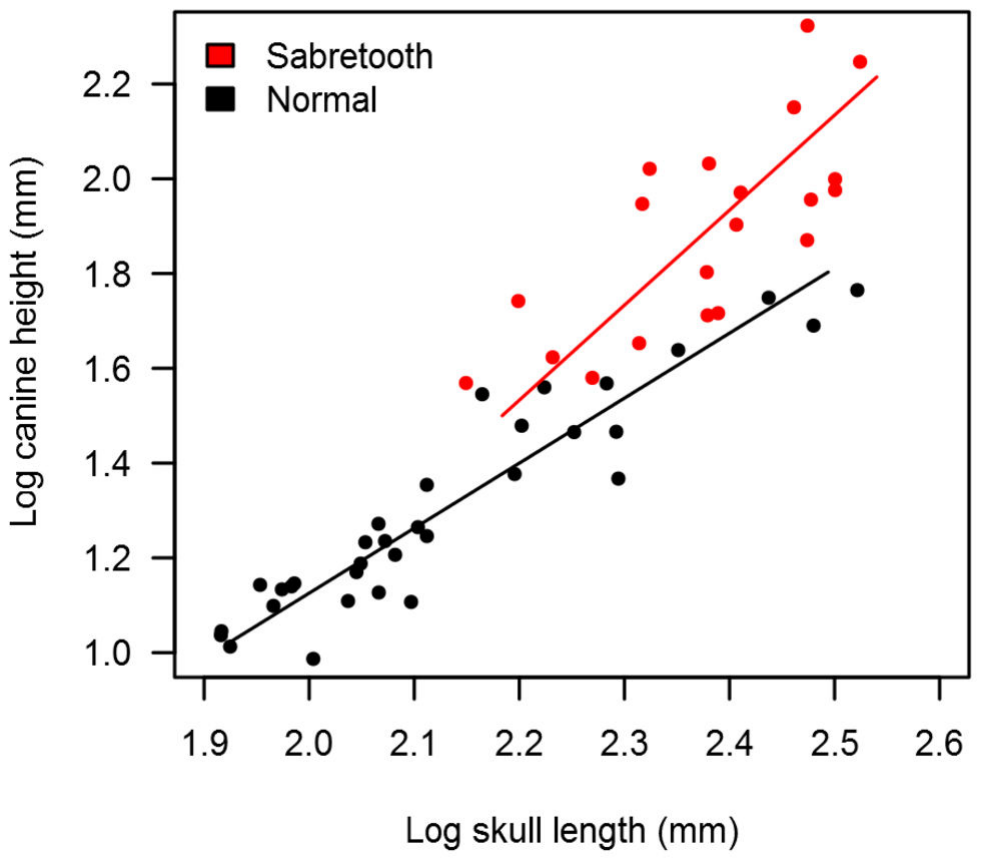
Regression of canine height against skull length. Regressions between changes in upper canine height versus skull length for sabretooths (red) and normal (i.e. non-sabretoothed) felids (black). Equations are given in the text.

**Figure 2:**
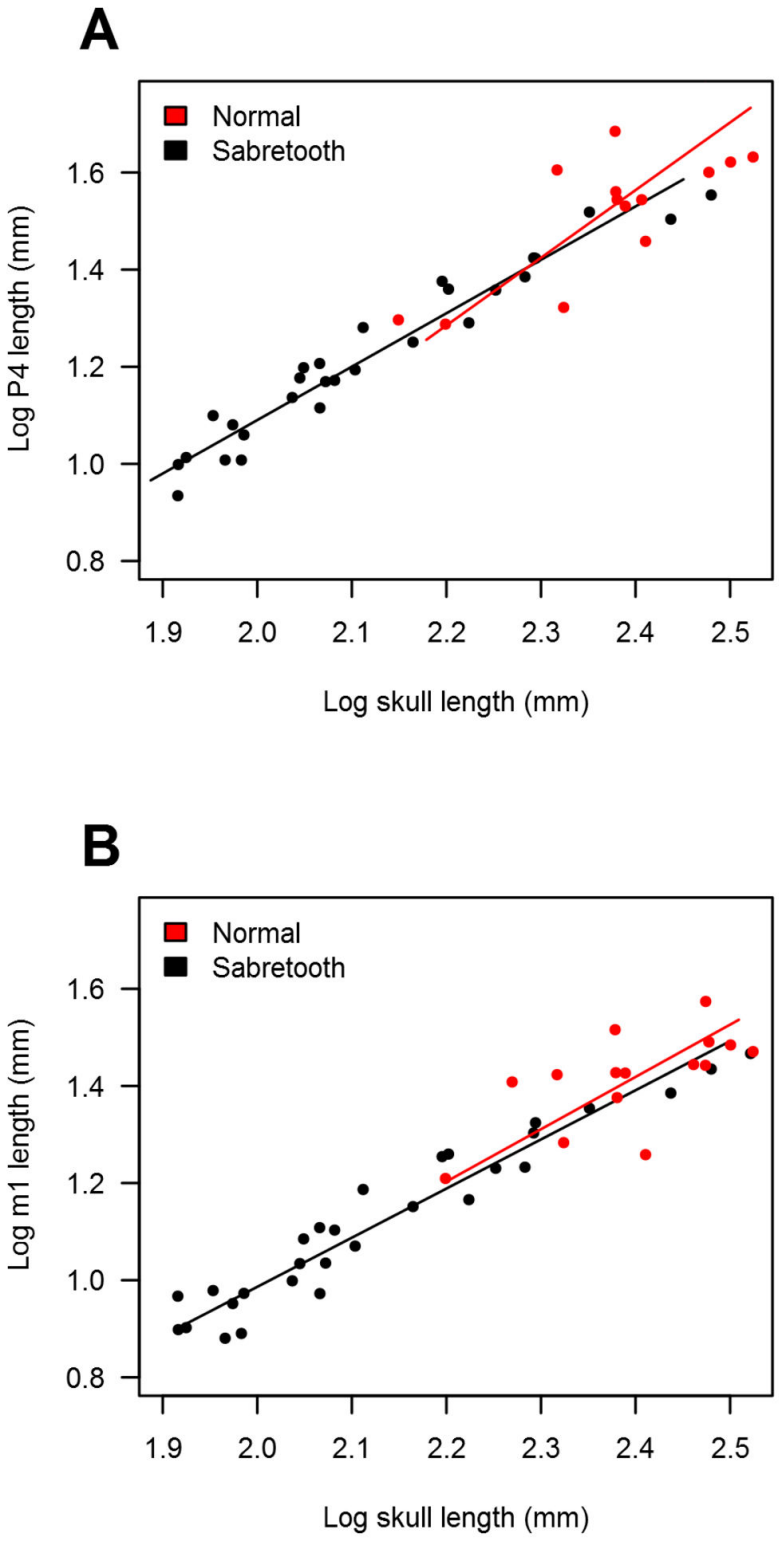
Regression of upper carnassial length and lower carnassial length against skull length. Regression between changes in upper carnassial length (A) and lower carnassial length (B) versus skull length for sabretooths (red) and normal (i.e. non-sabretoothed) felids (black). Equations are given in the text.

All upper canine measurement regressions against SL have slopes greater and statistically different than 1 for both sabretooths (logCH = 2.004*logSL - 2.877, *p*<0.001, n=20; logCW = 1.581*logSL - 2.665, *p*<0.009, n=9; logCL = 1.603*logSL - 2.392, *p*<0.001, n=17) and non-sabretooths (logCH = 1.371*logSL - 1.617, *p*<0.001, n=34; logCW = 1.393*logSL - 2.131, *p*<0.001, n=11; logCL = 1.348*logSL - 1.923, *p*<0.001, n=29). Slopes for sabretooths and non-sabretooths were statistically different from each other (*p*=0.033) for regression of CH against SL, although this was not the case for CW against SL (*p*=0.440) or CL against SL (*p*=0.181) (Figure 1). Slopes for dirk-tooths and scimitar-tooths were instead not statistically different from each other for regression of CH against SL (*p*=0.116), CW against SL (*p*=0.834), or CL against SL (*p*=0.320). Conversely, regressions of carnassial length against SL had slopes that were not statistically different from 1 in either sabretooths (logP4L = 1.393*logSL -1.780, *p*>0.05, n=14; logm1L = 1.077*logSL -1.167, *p*>0.05, n=15) or non-sabretooths (logP4L = 1.100*logSL -1.110, *p*>0.05, n=28; logm1L = 1.012*logSL -1.037, *p*>0.05, n=29), and the slopes were not statistically different from each other for either the PM4 regressions (*p*=0.229) or m1 regressions (*p*=0.775) (Figure 2).

## Discussion

Whereas carnassial teeth of both sabretooths and non-sabretoothed felids scale isometrically with skull length, supporting their close relationship with meat-slicing and an absence of associated sexual selection [2,10,11,23], upper canine height, width and length in both groups all show positive allometry. Although research into allometry has produced several different models to explain scaling relationships that are not reliant on sexual selection (e.g. elastic similarity, stress similarity) [26–29], all of these models predict scaling exponents less than one, and so are unlikely to be relevant to our findings. Instead, these results suggest that sexual selection rather than natural selection may have contributed to upper canine evolution across the Feliformia. Indeed, whereas canine morphology in living felids is undeniably closely associated with prey capture [1,2,10], intraspecific ethological interactions associated with upper canines (both agonistic visual display and male-male fighting) have also been recorded in many species [30], supporting the contributing role of sexual selection in the evolution of these teeth. Sexual dimorphism, a characteristic often associated with sexual selection, is also present in upper canine size among large-bodied and small-bodied felids [30,31]. Although we did not detect any statistically significant difference in the allometry of upper canine height between dirk-toothed and scimitar-toothed sabretooth ecomorphs, the statistically significant stronger positive allometry shown by this dental measurement in sabretooths as a whole compared to non-sabretooths further suggests that canine morphology in these extinct carnivores was driven by sexual selection to a greater extent. Upper canine height represents the tooth dimension with greatest external visibility, and so may be most sensitive to sexual selection for display.

We cannot exclude the hypothesis that natural selection was also an important associated factor responsible for driving and shaping the repeated evolution of the sabretooth morphotype in mammalian carnivores. Prey body mass in living mammalian carnivores displays positive allometry [32], suggesting that stronger positive allometry of upper canine height in sabretooths may be associated with natural selection through an evolutionary shift to larger-bodied ‘megaherbivore’ prey; the upper canines of sabretooths may therefore have become differentiated in size to permit optimal capture of different prey species and reduce interspecific competition [23,31], a process that has also been documented in extant carnivores [31,33,34]. Carnivores also display positive allometry and decreased disparity in other morphological traits associated with shifts in diet, with an increase in predator body size above a threshold limit of c.25kg driven by an energetic requirement for larger-bodied prey, and anatomical switching in the shape of the forelimb humerus-radius/humerus-ulna joint between either cursorial/pursuit or ambush modes in response to strong natural selection forces [35–37]. If driven by natural rather than sexual selection, the slope of the sabretooth upper canines would therefore represent one of the few examples of positive allometry in carnivore morphology associated with natural selection (see also 38). Furthermore, although our analyses did not detect any statistically significant differences in allometry of upper canine height between dirk-toothed and scimitar-toothed sabretooth ecomorphs, it is possible that different combinations of selective forces might have acted on different higher-order sabretooth taxa, notably the nimravids, some of which (e.g. *Nimravus*) had an ‘intermediate’ dental condition with canines that were shorter and less compressed than in other sabretooths [39]; this could represent a fruitful subject for further study.

However, although differences in overall morphology were undoubtedly associated with different selective pressures acting on the three different sabretooth ecomorphs and higher-order taxa, our analyses of dirk-tooths versus scimitar-tooths support the suggestion of previous authors [40] that it is unlikely that such adaptive differences were also associated with completely disparate functions for the elongated canines, so that it is still appropriate to consider these taxa and ecomorphs together as an evolutionarily coherent ‘sabretooth morphotype’. Furthermore, although the specific mechanism(s) by which different sabretooths captured and processed prey remains the subject of ongoing investigation [1,2,4–6,11], and may have varied between different sabretooth ecomorphs [17], recent biomechanical analysis suggests that hypertrophied anterior dentition is increasingly inefficient for killing larger-bodied prey [7], supporting our interpretation of sexual selection as an important evolutionary driver for the sabretooth morphotype. Similarly, although extinct sabretooths show minimal sexual dimorphism in craniodental proportions [22], sexual dimorphism is not a prerequisite for sexual selection, as sexually selected characters may be present in both males and females [41–43].

The evolution of many morphological characters has been shaped by both natural selection and sexual selection [44]. Indeed, the specialised sabretooth anterior dentition must still have permitted these carnivores to feed efficiently, and so the expression of these structures would have been ultimately constrained by strong natural selection. However, although the evolutionary drivers of many morphological structures in extinct species may always remain enigmatic, scaling relationships indicate that sexual selection is likely to have played a more important role in the repeated evolution of the hypertrophied sabretooth anterior dentition than has previously been recognized.

## Supporting Information

Table S1 **Morphometric data for species used in this study. Description of traits and measurements given in main text.** Category: S = sabretooth, NS = non-sabretooth.
(DOCX)
