# Supplementary material for: Canine Evolution in Sabretoothed Carnivores: Natural Selection or Sexual Selection?
Source: PLoS One. 2013 Aug 8;8(8):e72868. doi: 10.1371/journal.pone.0072868 (PMC3738559; doi:10.1371/journal.pone.0072868)
Supplement: Table S1 — Morphometric data for species used in this study. Description of traits and measurements given in main text. Category: S = sabretooth, NS = non-sabretooth. (DOCX) [file pone.0072868.s001.docx]

**Table S1.** Morphometric data for species used in this study. Description of traits and measurements given in main text. Category: S = sabretooth, NS = non-sabretooth.

| **Family** | **Species** | **Category** | **Skull length (mm)** | **Canine crown height (mm)** | **Canine length (mm)** | **P4 length**  **(mm)** | **m1 length**  **(mm)** |
| --- | --- | --- | --- | --- | --- | --- | --- |
| Felidae: Felinae | *Catopuma temminckii* | NS | 113.10 | 17.10 | — | — | — |
| Felidae: Felinae | *Felis chaus* | NS | 96.80 | 14.00 | 5.19 | 11.48 | 9.40 |
| Felidae: Felinae | *Felis silvestris* | NS | 92.47 | 12.56 | 4.87 | 10.19 | 7.60 |
| Felidae: Felinae | *Oncifelis geoffroyi* | NS | 94.20 | 13.60 | 5.79 | 12.04 | 8.95 |
| Felidae: Felinae | *Pardofelis marmorata* | NS | 89.80 | 13.90 | 5.92 | 12.57 | 9.52 |
| Felidae: Felinae | *Prionailurus bengalensis* | NS | 82.50 | 11.10 | 4.49 | 9.97 | 7.91 |
| Felidae: Felinae | *Prionailurus planiceps* | NS | 96.20 | 13.80 | 4.62 | 10.18 | 7.77 |
| Felidae: Felinae | *Prionailurus viverrinus* | NS | 118.10 | 17.20 | 7.70 | 14.77 | 10.85 |
| Felidae: Felinae | *Neofelis diardi* | NS | 146.10 | 35.10 | 10.93 | 17.82 | 14.18 |
| Felidae: Felinae | *Lynx rufus* | NS | 108.89 | 12.85 | 6.47 | 13.70 | 9.97 |
| Felidae: Felinae | *Lynx canadensis* | NS | 116.40 | 18.70 | 7.37 | 16.10 | 12.83 |
| Felidae: Felinae | *Lynx lynx* | NS | 129.40 | 22.60 | 9.47 | 19.09 | 15.38 |
| Felidae: Felinae | *Lynx pardinus* | NS | 120.73 | 16.09 | 7.29 | 14.86 | 12.68 |
| Felidae: Felinae | *Hepailurus yaguarondi* | NS | 100.90 | 9.71 | 5.79 | — | — |
| Felidae: Felinae | *Felis aurata* | NS | 111.94 | 15.42 | 7.58 | 15.78 | 12.16 |
| Felidae: Felinae | *Felis temminckii* | NS | 125.03 | 12.79 | — | — | — |
| Felidae: Felinae | *Felis viverrina* | NS | 129.42 | 17.62 | — | — | — |
| Felidae: Felinae | *Leptailurus serval* | NS | 116.47 | 13.40 | 6.19 | 13.04 | 9.39 |
| Felidae: Felinae | *Leopardus wiedii* | NS | 82.41 | 10.89 | 5.65 | 8.60 | 9.27 |
| Felidae: Felinae | *Leopardus pardalis* | NS | 126.90 | 18.40 | 8.75 | 15.62 | 11.76 |
| Felidae: Felinae | *Leopardus tigrinus* | NS | 84.10 | 10.30 | 4.24 | 10.31 | 7.99 |
| Felidae: Felinae | *Neofelis nebulosa* | NS | 167.40 | 36.31 | 12.56 | 19.52 | 14.65 |
| Felidae: Felinae | *Caracal caracal* | NS | 110.92 | 14.79 | 6.91 | 15.03 | 10.82 |
| Felidae: Felinae | *Uncia uncia* | NS | 156.85 | 23.82 | 10.74 | 23.77 | 17.97 |
| Felidae: Felinae | *Puma concolor* | NS | 178.70 | 29.20 | 12.19 | 22.80 | 17.00 |
| Felidae: Felinae | *Acinonyx jubatus* | NS | 159.30 | 30.13 | 14.79 | 22.90 | 18.19 |
| Felidae: Felinae | *Panthera onca* | NS | 224.50 | 43.50 | 15.07 | 33.00 | 22.60 |
| Felidae: Felinae | *Panthera pardus* | NS | 191.90 | 37.00 | 13.94 | 24.29 | 17.09 |
| Felidae: Felinae | *Panthera leo* | NS | 302.00 | 49.00 | 23.09 | 35.79 | 27.22 |
| Felidae: Felinae | *Panthera tigris* | NS | 273.70 | 56.10 | 23.93 | 31.91 | 24.29 |
| Felidae: Felinae | *Miracinonyx inexpectatus* | NS | 196.00 | 29.25 | 14.65 | 26.55 | 20.10 |
| Felidae: Felinae | *Acinonyx pardinensis* | NS | 196.90 | 23.30 | 13.90 | 26.50 | 21.10 |
| Felidae: Felinae | *Panthera atrox* | NS | 332.40 | 58.21 | — | — | 29.30 |
| Felidae: Machairodontinae | *Nimravides galiani* | S | 245.00 | 52.05 | 24.60 | 33.95 | 26.70 |
| Felidae: Machairodontinae | *Dinofelis barlowi* | S | 239.40 | 51.50 | 24.38 | 36.35 | 26.75 |
| Felidae: Machairodontinae | *Smilodon gracilis* | S | 240.16 | 107.65 | 26.06 | 35.03 | 23.77 |
| Felidae: Machairodontinae | *Smilodon fatalis* | S | 289.30 | 141.50 | 44.26 | — | 27.80 |
| Felidae: Machairodontinae | *Smilodon populator* | S | 334.40 | 176.50 | 50.12 | 42.85 | 29.57 |
| Felidae: Machairodontinae | *Homotherium crenatidens* | S | 300.30 | 90.37 | 32.27 | 39.86 | 30.95 |
| Felidae: Machairodontinae | *Homotherium serum* | S | 297.80 | 74.20 | 34.59 | — | 27.70 |
| Felidae: Machairodontinae | *Machairodus aphanistus* | S | 316.60 | 99.80 | — | — | — |
| Felidae: Machairodontinae | *Machairodus giganteus* | S | 316.60 | 94.62 | 36.81 | 41.83 | 30.51 |
| Felidae: Machairodontinae | *Megantereon cultridens* | S | 257.40 | 93.50 | 21.18 | 28.73 | 18.14 |
| Felidae: Machairodontinae | *Paramachairodus ogygia* | S | 170.50 | 42.00 | 15.03 | — | — |
| Felidae: Machairodontinae | *Ischyrosmilus* sp. | S | 255.00 | 80.00 | 31.00 | 35.00 | — |
| Nimravidae | *Eusmilus sicarius* | S | 210.86 | 104.95 | 31.99 | 21.00 | 19.20 |
| Nimravidae | *Dinictis felina* | S | 141.00 | 37.07 | 13.61 | 19.80 | — |
| Nimravidae | *Hoplophoneus primaevus* | S | 158.13 | 55.21 | 14.59 | 19.40 | 16.20 |
| Nimravidae | *Nimravus debilis* | S | 186.00 | 38.02 | 16.00 | — | 25.60 |
| Nimravidae | *Nimravus gomphodus* | S | 206.06 | 44.98 | 16.00 | — | — |
| Barbourofelidae | *Barbourofelis morrisi* | S | 207.49 | 88.51 | 26.18 | 40.30 | 26.50 |
| Barbourofelidae | *Barbourofelis fricki* | S | 298.00 | 210.38 | — | 63.00 | 37.50 |
| Barbourofelidae | *Barbourofelis loveorum* | S | 239.00 | 63.53 | — | 48.40 | 32.80 |

**ESM Text S1.** Natural History Museum specimens used for further craniodental morphometric measurements.

*Acinonyx jubatus* (NHM 1926.6.4.5, 1926.6.4.4, 1931.1.3.3, 35.2.3.1, 1905.11.5.10)

*Felis aurata* (NHM 25.10.7.13, 24.8.8.1, 30.12.15.10, 1.11.21.6, 1938.3.29.1)

*F. chaus* (NHM 1172/cl, 6.1.2.3, 4.6.1.1, 8.1.13.4, 53.1.6.86)

*F. marmorata* (NHM 46.6.15.7, 79.1634, 42.2.15.235, 55.1645, 26.10.4.49)

*F. silvestris* (NHM 45.44, 4.1.25.5, 45.30, 45.22, 45.32)

*Leopardus pardalis* (NHM 1905.5.4.3, 1882.10.26.9, 1894.12.18.3, 10.9.29(12), 1913.12.12.1)

*L. tigrinus* (NHM 76.665, 1884.2.8.3, 28.10.11.4, 3.7.1.11, 22.5.19.6)

*Leptailurus serval* (NHM 41.3.17.17, 34.2.24.27, 9.11.2.3, 31.11.1.4, 66.795)

*Lynx canadensis* (NHM 92.4.19.1, 63.2.24.40, 68.12.29.34, 1.6.29.1, 62.12.12.29)

*L. lynx* (NHM 83.4.21.1, 98.8.5.1, 1896.10.19.16, 51.11.8.16, 55.12.26.176)

*L. pardinus* (NHM 55.12.26.179, 95.9.4.1, 72.10.26.1, 7.6.4.2, 39.3664)

*Neofelis diardi* (NHM 1938.11.30.23, 1903.4.9.2, 40.377, 1938.11.30.22, 39.336)

*N. nebulosa* (NHM 58.6.24.49, 47.685, 43.65, 54.7.3.1, 30.3.3.4)

*Oncifelis geoffroyi* (NHM 12.5.4.1, 24.9.1.1, 1924.9.1.2, 9.12.1.9, 1917.1.25.2)

*Panthera leo* (NHM 35.2.14.3, 76.202, 61.447, 25.6.17.10, 36.5.26.6)

*P. tigris* (NHM 39.335, 1934.12.1.1, 20.11.14.2, 39.335, 36.5.26.2)

*Prionailurus bengalensis* (NHM 40.380, 51.186, 14.8.22.18, 32.3.1.21, 21.8.2.6)

*P. planiceps* (NHM 64.8.17.7, 75.2246, 1980.1n.130.a, 46.4.10.8)

*P. viverrinus* (NHM 8.11.1.8, 10.3.10.2, 50.1495, 1938.11.30.34, 50.1495)

*Uncia uncia* (NHM 75.2283, 33.7.20.2, 67.3.20.1, 81.14.98, 1938.6.28.3)

**ESM References**

Antón M, Galobart A, Turner A (2005) Co-existence of scimitar-toothed cats, lions and hominins in the European Pleistocene. Implications of the post-cranial anatomy of *Homotherium latidens* (Owen) for comparative palaeoecology. Quat Sci Rev 24: 1287-1301.

Baskin J (1981) *Barbourofelis* (Nimravidae) and *Nimravides* (Felidae), with a description of two new species from the Late Miocene of Florida. J Mammal 62: 122-139.

Christiansen P (2007) Canine morphology in the larger Felidae: implications for feeding ecology. Biol J Linn Soc 91: 573-592.

Christiansen P (2007) Comparative bite forces and canine bending strength in feline and sabretooth felids: implications for predatory ecology. Zool J Linn Soc 151: 423-437.

Christiansen P (2008) Evolution of skull and mandible shape in cats (Carnivora: Felidae). PLoS ONE 3(7): e2807.

Christiansen P, Adolfssen JS (2005) Bite forces, canine strength and skull allometry in carnivores. J Zool 266: 133-151.

Christiansen P, Harris JM (2005) Body size of *Smilodon* (Mammalia: Felidae). J Morphol 266: 369-384.

Christiansen P, Harris JM (2009) Craniomandibular morphology and phylogenetic affinities of *Panthera atrox*: implications for the evolution and paleobiology of the lion lineage. J Vert Paleont 29: 934-945.

Farlow JO, Pianka ER (2003) Body size overlap, habitat partitioning and living space requirements of terrestrial vertebrate predators: implications for the paleoecology of large theropod dinosaurs. Hist Biol 16: 21-40.

Gittleman JL, Van Valkenburgh B (1997) Sexual dimorphism in the canines and skulls of carnivores: effects of size, phylogeny, and behavioural ecology. J Zool 242: 97-117.

Kurten B (1978) The lynx from Etouaires, *Lynx issiodorensis* (Croizet & Jobert), late Pleiocene. Ann Zool Fennici 15: 314-322.

Martin LD, Babiarz JP, Naples VL, Hearst J (2000) Three ways to be a saber-toothed cat. Naturwissenschaften 87: 41-44.

O’Regan HJ, Reynolds SC (2009) An ecological reassessment of the southern African carnivore guild: a case study from Member 4, Sterkfontein, South Africa. J Hum Evol 57: 212-222.

Palmqvist P, Arribas A, Martinex-Navarro B (1999) Ecomorphological study of large canids from the lower Pleistocene of southeastern Spain. Lethaia 32: 75-88.

Peigne S (2001) A primitive nimravine skull from the Quercy fissures, France: implications for the origin and evolution of Nimravidae (Carnivora). Zool J Linn Soc 132: 401-410.

Pertoldi C, García-Perea R, Godoy JA, Delibes M, Loeschcke V (2006) Morphological consequences of range fragmentation and population decline on the endangered Iberian lynx (*Lynx pardinus*). J Zool 268: 73-86.

Peters G, Baum L, Peters MK, Tonkin-Leyhausen B (2009) Spectral characteristics of intense mew calls in cat species of the genus *Felis* (Mammalia: Carnivora: Felidae). J Ethol 27: 221-237.

Salesa MJ, Anton M, Turner A, Morales J (2005) Aspects of the functional morphology in the cranial and cervical skeleton of the sabre-toothed cat *Paramachairodus ogygia* (Kaup, 1832) (Felidae, Machairodontinae) from the Late Miocene of Spain: implications for the origins of the machairodont killing bite. Zool J Linn Soc 144: 363-377.

Salesa MJ, Anton M, Turner A, Morales J (2006) Inferred behaviour and ecology of the primitive sabre-toothed cat *Paramachairodus ogygia* (Felidae, Machairodontinae) from the Late Miocene of Spain. J Zool 268: 243–254.

Slater GJ, Van Valkenburgh B (2008) Long in the tooth: evolution of sabertooth cat cranial shape. Paleobiol 34: 403-419.

Turner A, Antón M (1997) The big cats and their fossil relatives. New York: Columbia University Press. 254 pp.

Van Valkenburgh B (1988) Trophic diversity in past and present guilds of large predatory mammals. Paleobiol 14: 155-173.

Van Valkenburgh B (1989) Carnivore dental adaptations and diet: a study of trophic diversity within guilds. In: Gittleman JL, editor. Carnivore Behavior, Ecology and Evolution. New York: Cornell University Press. pp. 410-436.

Van Valkenburgh B, Grady F, Kurten B (1990) The Plio-Pleistocene cheetah-like cat *Miracinonyx inexpectatus* of North America. J Vert Paleont 10: 434-454.

Van Valkenburgh B, Ruff C B (1987) Canine tooth strength and killing behaviour in large carnivores. J Zool 212: 379–397.

Werdelin L, Lewis ME (2001) A revision of the genus *Dinofelis* (Mammalia, Felidae). Zool J Linn Soc 132: 147-258.

Wroe S, McHenry C, Thomason J (2005) Bite club: comparative bite force in big biting mammals and the prediction of predatory behaviour in fossil taxa. Proc Roy Soc B 272: 619-625.

Wroe S, Milne N (2007) Convergence and remarkably consistent constraint in the evolution of carnivore skull shape. Evol 61: 1251-1260.

Yensen E, Seymour KL (2000) *Oreailurus jacobita*. Mammal. Species 644: 1-6.
